# A pharmacogenomic approach to the treatment of children with GH deficiency or Turner syndrome

**DOI:** 10.1530/EJE-13-0069

**Published:** 2013-09

**Authors:** P Clayton, P Chatelain, L Tatò, H W Yoo, G R Ambler, A Belgorosky, S Quinteiro, C Deal, A Stevens, J Raelson, P Croteau, B Destenaves, C Olivier

**Affiliations:** 1 Manchester Academic Health Sciences Centre, Royal Manchester Children's Hospital5th Floor Research, Oxford Road, Manchester, M13 9WLUK; 2 Département PediatrieHôpital Mère-Enfant, Université Claude BernardLyonFrance; 3 Clinica PediatricaUniversità degli Studi di VeronaVeronaItaly; 4 Department of PediatricsAsan Medical Center, University of Ulsan College of MedicineSeoulRepublic of Korea; 5 The Children's Hospital at Westmead, University of SydneySydney, New South WalesAustralia; 6 Endocrine ServiceHospital de Pediatría GarrahanBuenos AiresArgentina; 7 Servicio de Pediatría EndocrinoHospital Materno InfantilLas Palmas de Gran CanariaSpain; 8 Department of PediatricsCHU-Sainte Justine, University of MontrealMontréal, QuebecCanada; 9 Genizon BioSciencesSt Laurent, QuebecCanada; 10 Merck Serono S.A.GenevaSwitzerland

## Abstract

**Objective:**

Individual sensitivity to recombinant human GH (r-hGH) is variable. Identification of genetic factors contributing to this variability has potential use for individualization of treatment. The objective of this study was to identify genetic markers and gene expression profiles associated with growth response on r-hGH therapy in treatment-naïve, prepubertal children with GH deficiency (GHD) or Turner syndrome (TS).

**Design:**

A prospective, multicenter, international, open-label pharmacogenomic study.

**Methods:**

The associations of genotypes in 103 growth- and metabolism-related genes and baseline gene expression profiles with growth response to r-hGH (cm/year) over the first year were evaluated. Genotype associations were assessed with growth response as a continuous variable and as a categorical variable divided into quartiles.

**Results:**

Eleven genes in GHD and ten in TS, with two overlapping between conditions, were significantly associated with growth response either as a continuous variable (seven in GHD, two in TS) or as a categorical variable (four more in GHD, eight more in TS). For example, in GHD, *GRB10* was associated with high response (≥*Q*3; *P*=0.0012), while *SOS2* was associated with low response (≤*Q*1; *P*=0.006), while in TS, *LHX4* was associated with high response (*P*=0.0003) and *PTPN1* with low response (*P*=0.0037). Differences in expression were identified for one of the growth response-associated genes in GHD (*AKT1*) and for two in TS (*KRAS* and *MYOD1*).

**Conclusions:**

Carriage of specific growth-related genetic markers is associated with growth response in GHD and TS. These findings indicate that pharmacogenomics could have a role in individualized management of childhood growth disorders.

## Introduction

Recombinant human GH (r-hGH) has proved to be a safe and effective treatment to increase growth rate and adult height across a range of growth disorders, and improve metabolic status in adult GH deficiency (GHD) (1, 2). There is, however, substantial interindividual variability in growth response to r-hGH (1), and health economic assessments have shown that variability in response to r-hGH is the most important factor determining the cost-effectiveness of treatment (3, 4, 5). Licensing authorities recognize that the posology of r-hGH needs to be individualized. Predicting growth response to r-hGH and personalizing dosing should, therefore, be a clinical priority. Beyond conventional growth predictors (e.g. age, weight and r-hGH dose at the start of treatment), the identification of genetic factors contributing to this variability can be used to promote individualization of GH (r-hGH) treatment for best patient outcome.

There are many pathways that regulate human growth, which include hormones, growth factors and cellular growth processes (6, 7). Polymorphisms that could alter the function of the genes in these pathways may affect growth response to r-hGH therapy. One such example is the GH receptor polymorphism, in which exon 3 is either present or absent. This has been shown to influence GH signal transduction *in vitro* and growth response to r-hGH *in vivo* (8): in meta-analyses, those who carry the exon 3 deletion grow more (by ∼1 cm in the first year) in response to r-hGH (9, 10). However, these meta-analyses demonstrate significant variation between reports on one condition, and between conditions. This highlights the limitations of studying the effect of a single gene on a complex trait, such as growth. Another approach to assessing r-hGH responsiveness, which uses a whole genome rather than candidate gene methodology, is to examine gene expression profiles. Using peripheral blood mononuclear cells (PBMCs) as the RNA source, this has generated data relevant to growth responses to r-hGH in children with GHD and Turner syndrome (TS), and in adults to detect r-hGH doping (11, 12). To date, a large-scale study in children with growth disorders has not been undertaken to address this important issue.

Pharmacogenomics has been successfully used in the field of cancer to identify benign vs malignant tumors and to quantify the risk of tumor recurrence (13, 14). Testing of specific genes is being used increasingly to predict response to drugs: the results of such tests can indicate whether a drug should not be used because of the risk of adverse events, or whether the dose to achieve a safe and efficacious outcome should be modified (15). In some instances, genetic testing has become part of the license requirements issued by regulatory authorities for use of a drug (15).

The PREDICT study (NCT00256126; Merck Serono S.A., Study 24531: A Phase IV Open-label Study of Predictive Markers in Growth Hormone Deficient and Turner Syndrome Pre-pubertal Children Treated with SAIZEN®) was a month-long trial to identify the most responsive serum biomarkers associated with growth response to r-hGH therapy. Two conditions, associated with significant short stature and well-characterized growth responses to r-hGH, were assessed, namely GHD and TS, which together account for ∼50% of r-hGH prescriptions.

The study presented here (NCT00699855; Merck Serono S.A., Study 28614: Observational Long-term Follow-up of the Phase IV Open-label Trial of Predictive Markers in Growth Hormone-Deficient and Turner Syndrome Pre-pubertal Children Treated with SAIZEN®) constitutes the first-year results following on from the PREDICT study, which uses a pharmacogenomic approach to evaluate the association of genetic polymorphisms in growth- and metabolism-related genes and baseline gene expression profiles using whole blood mRNA with long-term changes in growth while on r-hGH therapy. The objective of this study was to identify genetic markers and gene expression profiles associated with growth response (cm) 1 year after the initiation of r-hGH therapy in treatment-naïve prepubertal children with GHD or TS. This study demonstrates that a broad range of genes, related in particular to cell signaling, are associated with growth response to r-hGH. It also shows that the associated genes differ between GHD and TS, and that these genetic markers and expression profiles are associated with high or low first-year growth responses to r-hGH in children with GHD or TS. This work indicates that pharmacogenomics could be used to predict a key outcome of r-hGH treatment.

## Subjects and methods

### Study design

This open-label, prospective study involved three steps. First, candidate genes involved in growth and metabolism were identified by a literature search and selected for inclusion based on advice from a board of growth experts (see online Supplementary Table 1, see section on supplementary data given at the end of this article for a list of the candidate genes). Then, individual genotypes were assessed for their effect on growth (using full genotype, as well as dominant and recessive models for carriers of major and minor alleles), and associated markers were identified. Finally, the predictive potential of these markers was evaluated by categorizing the patient population into three groups based on height change in three age bands (<8, 8–12 and >12 years): high (>75th percentile (≥*Q*3)), intermediate (between the 25th and 75th percentiles (>*Q*1–<*Q*3)) and low (<25th percentile (≤*Q*1)). Analysis was carried out separately for patients with GHD and TS.

This study was conducted in compliance with ethical principles based on the Declaration of Helsinki, the International Conference on Harmonization Tripartite Guideline for Good Clinical Practice, and all applicable regulatory requirements.

### Patients and treatment interventions

In total, 170 patients (110 with GHD and 60 with TS) underwent a genetic analysis from a per-protocol population at 1 year of 182 patients (115 with GHD and 67 with TS). The patients were recruited in 14 countries from across the world (listed under the Acknowledgements section). All the patients were prepubertal at the start of treatment. The diagnosis of GHD was based on two different stimulation tests with a peak GH value <10 μg/l, using assays in the local center. Patients selected for r-hGH treatment were based on criteria used in the local units. Patients with GHD associated with etiologies such as CNS tumors with or without cranial irradiation were excluded. The median peak GH value was 4.1 μg/l (Table 1), and <25% of the patients had a value >5.6 μg/l, and only eight patients had a peak GH value between 7 and 10 μg/l. TS required karyotype confirmation. Patients with GHD received r-hGH at an average dose of 0.035 mg/kg per day, and patients with TS received an average dose of 0.051 mg/kg per day. Other hormone deficiencies (cortisol and thyroxine), if present, were appropriately treated. Compliance was monitored by recall in the last month of the study, and was estimated at an average of ∼90% in both conditions.

Growth parameters were converted to SDS using the Sempé reference data (16), so that all children were compared with the same standard. Baseline insulin-like growth factor 1 (IGF1) and IGF binding protein 3 (IGFBP3) were measured in a central laboratory (qLAB, Livingston, Edinburgh, UK), using the DPC chemiluminescent immunoassay (Immunolite 2000; Siemens Healthcare Diagnostics, Norwood, MA, USA). Levels were converted to SDS using relevant reference data (17). Baseline characteristics are shown in Table 1.

### Genetic analysis

Genotyping was performed centrally on DNA extracted from whole blood using an Illumina GoldenGate micro array, containing 1536 single nucleotide polymorphisms (SNPs) located in 103 candidate genes related to i) the GH/IGF1 axis, ii) bone and cell growth and iii) glucose and lipid metabolisms. A total of 1451 SNPs were successfully genotyped. Prior to analysis, the genotyping data for these SNPs were filtered to remove SNPs with low minor allele frequency (<10%), those with a call rate below 95%, and those (except for X-linked SNPs in girls with TS and boys with GHD) showing significant deviation from the Hardy–Weinberg equilibrium using a Bonferroni correction for 1451 tests. After this cleaning step, 1182 SNPs in patients with GHD and 1183 SNPs in patients with TS remained for analysis. All analyses were performed centrally by the Bioinformatics Group at Merck Serono.

Gene expression profiling was carried out on whole blood RNA extracted centrally by qLAB using the PAXgene 96 blood RNA Kit (Qiagen) at baseline: 67 samples were available for GHD and 44 for TS. Reduction of globin mRNA was undertaken using the Ambion GLOBIN clear Human Kit (Life Technologies). The quality of RNA was assessed using an ND-1000 spectrophotometer (NanoDrop, Wilmington, DE, USA) and qualified using an Agilent 2100 Bioanalyzer (Agilent Technologies, Santa Clara, CA, USA). cRNA was generated using the Two-Cycle Eukaryotic Target Labeling Kit (Affymetrix, Santa Clara, CA, USA) and a final quality check was performed before hybridization to Affymetrix GeneChip Human Genome U133 Plus 2.0 Arrays. Arrays were then scanned on an Affymetrix GeneChip 3000 7G scanner and assessed for quality against internal and hybridization controls. All analyses were performed centrally by the Bioinformatics Group at Merck Serono.

Processing and normalization of the raw gene expression data were performed on GHD and TS samples using a Robust Multi-array Average background correction modified for probe sequence with quantile normalization and median polish (Partek Genomics Suite version 6.3, Partek Inc., St. Louis, MO, USA). Outliers were identified by cross-validation using principal component analysis (PCA) and Isomap multidimensional scaling (Qlucore Omics Explorer 2.2, Qlucore AB, Lund, Sweden) to generate first-year growth response datasets in GHD and TS. A variance cut-off relative to the variable with the largest variance (*σ*max) was used to remove noninformative probes; this was set at 0.05 *σ*/*σ*max (Qlucore Omics Explorer 2.2). All processing of array data was performed at the University of Manchester.

### Statistical analysis

#### Continuous analysis

SNPs associated with first-year growth were identified using the Kruskal–Wallis rank sum test on the genotype (additive model), the presence or the absence of the major allele (dominant model) and the presence or the absence of the minor allele (recessive model). For nonpseudoautosomal X-linked markers, boys with GHD were analyzed separately from girls with GHD. As a candidate gene, rather than a whole genome, approach was being used, both unadjusted *P* values and adjusted *P* values calculated using a Bonferroni correction that takes into account the number of linkage disequilibrium (LD) blocks present in the gene containing the SNP are reported for each SNP.

#### Categorical analysis

Markers were then tested in a second stage of the analysis, where patients were classified by quartiles, based on the normal distribution of growth response, as high (≥*Q*3), intermediate (>*Q*1–<*Q*3) or low responders (≤*Q*1) in each of three age groups (<8, 8–12 and >12 years) to control for the potential impact of age on response to r-hGH. Markers were assessed by comparing high responders vs intermediate+low responders, and low responders vs intermediate+high responders. All *P* values were calculated using Fisher's exact test and are shown as both unadjusted and Bonferroni-corrected values using the number of LD blocks within each candidate gene.

All demographic and growth data were analyzed by the Biostatistics Group at Merck Serono. Both the continuous and categorical analyses were conducted by Genizon BioSciences (Montreal, QC, Canada).

#### Country of origin analysis

In order to address whether country of origin or population stratification may be a confounding factor in response to r-hGH, a PCA was undertaken using the PLINK genetic analysis software (http://pngu.mgh.harvard.edu/∼purcell/plink/) by PGx Services. The genotypes for the 1182 GHD and 1183 TS SNPs were first screened to produce Tag SNPs that were in linkage equilibrium (*R*^2^<0.2 for LD between any two Tag SNPs). This was performed twice, independently, to generate lists of Tag SNPs for GHD and TS, on which PCA was carried out. The first two PCs were then used to assess impact on growth response.

#### Gene expression profiles

Gene expression associated with first-year growth response (cm) in GHD and TS was identified in low vs intermediate+high responders, and in high vs intermediate+low responders (as defined above) using ANOVA (*P*<0.05), with control for gender and age. Control for age was undertaken as we have recently shown that gene expression in healthy children is age dependent (18). In order to better understand the function and significance of these growth-associated genes, the analysis of inferred protein–protein interaction networks was performed using Ingenuity Pathways Analysis (IPA) Software. This allows differentially expressed genes to be correlated with biologic pathways. IPA was also used to generate inferred interaction networks derived from the genes associated with growth response. Gene expression data were then mapped onto these inferred networks to allow the integration of gene expression and genetic analyses, and to assess the presence of putative expression quantitative trait loci (eQTL). All analyses of array data were performed at the University of Manchester.

#### *In silico* evaluation of predicted function for significant SNPs

The predicted consequences of an SNP on transcriptional activity have been derived based on data from many different cell lines in which transcription factors and their binding sites responsible for modulating gene transcription, as identified by chromatin immunoprecipitation sequencing (ChIP-seq), are listed in the Encyclopaedia of DNA Elements (ENCODE) database (http://genome.ucsc.edu/ENCODE/) (19). Using this database, SNPs associated with growth response to r-hGH in this study, which lie in or near these binding sites and have been shown to have an impact on transcription, were identified.

## Results

### Genetic markers and expression profiles associated with height change in children with GHD

The children with GHD had a median basal growth rate of 4 (*Q*1, 3; *Q*3, 6) cm/year, and then grew a median of 8.5 (*Q*1, 7.3; *Q*3, 10.2) cm over the first year. Ten polymorphisms within seven different genes were found to be significantly associated with this growth response, assessed as a continuous trait (Table 2). These included the gene coding for the major GH-dependent carrier of IGF1 in the circulation, *IGFBP3*; signaling molecules *GRB10* and *SOS1* (MAPK pathway); the phosphatase *INPPL1*; the growth factor *TGF*α the tumor suppressor *TP53*; and *CYP19A1*, a P450 cytochrome enzyme with aromatase activity. For each polymorphism, the difference in growth between alleles or genotypes was >1 cm over the first year, representing ∼20% of first-year increment in growth.

**Figure 1:**
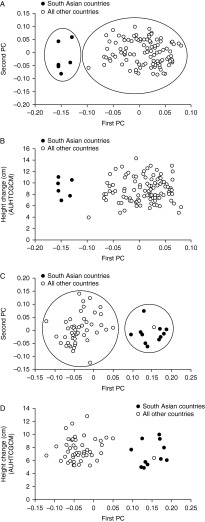
(A and C) The first and second principal components (PCs), based on a PC analysis undertaken on Tag single nucleotide polymorphisms for children with (A) GH deficiency (GHD) and (C) Turner syndrome (TS). The first PC clearly demarcates children from Asian countries vs children from all other countries. (B and D) The relationship between first-year growth response and the first PC in children with (B) GHD and (D) TS. There is complete overlap in growth response between children from Asian countries vs children from all other countries. The same overlap occurs with the second PC (data not shown). Countries are Argentina, Australia, Austria, Canada, France, Germany, Italy, Korea, Norway, Russia, Spain, Sweden, Taiwan and UK.

To control for the potential impact of age on growth response, genes associated with growth, defined as high (≥*Q*3), intermediate (>*Q*1–<*Q*3) or low responders (≤*Q*1) in each of three age groups, were identified. Four of the genes in the continuous trait analysis were also found by this categorical analysis (Table 3), while a further four genes were added: *IGFII* (*IGF2*), *CYR61* (a secreted protein, also known as IGFBP10), *AKT1* (a signaling molecule activated by PI3K) and *SOS2* (MAPK signaling). Importantly, the r-hGH doses between the high, intermediate and low responders did not differ (Table 4). To control for the potential impact of country of origin on response, a PCA was undertaken. The first principal component (PC) based on the Tag SNPs clearly separated those children from Asia from all other children (Fig. 1A). However, there was complete overlap in growth response between the groups (Fig. 1B).

**Figure 2:**
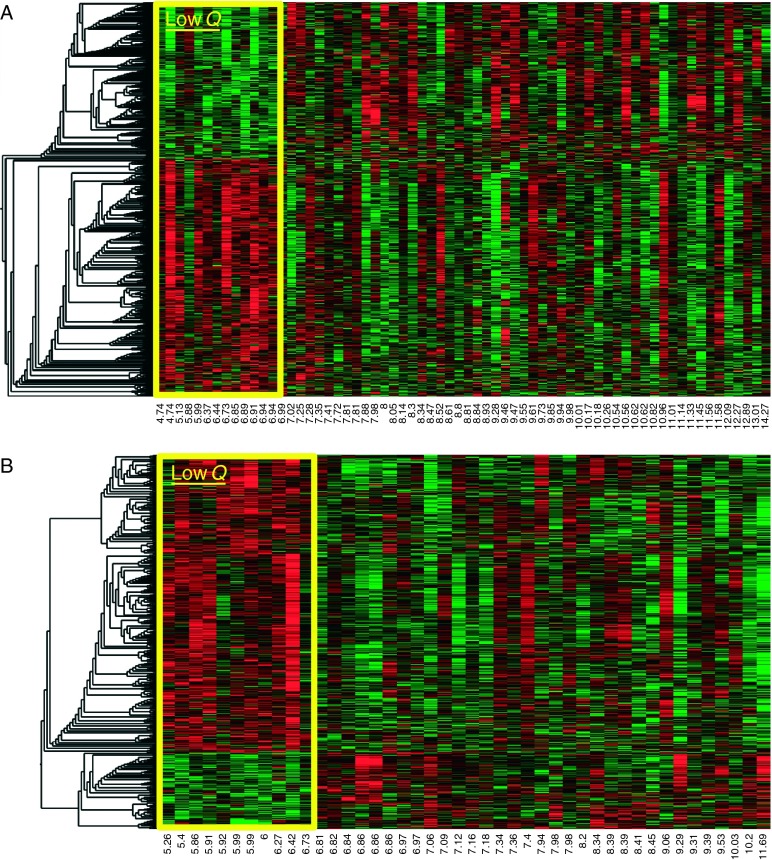
‘Heat map’ of genes associated with first-year growth response to r-hGH in (A) children with GH deficiency (GHD) and (B) girls with Turner syndrome (TS). Each column (*x*-axis) represents an individual child (with growth rate (cm) shown), and each row (*y*-axis) represents an individual gene. Red color in a cell indicates increased gene expression and green color in a cell represents decreased gene expression in the lowest quartile vs the rest. The box indicates the expression profiles of those in the lowest quartile. The ‘dendrogram’, which groups ‘clusters’ of genes with similar expression levels, is shown on the left side of each figure. Low *Q*, lowest quartile (≤*Q*1).

A total of 1886 gene expression probe sets corresponding to 1188 genes (Ingenuity Knowledge Base) were associated with first-year growth in the expression profiles for the low responder analysis (Fig. 2A): a distinct pattern of gene expression at baseline in the low responders compared with the other profiles was identified. A total of 1127 gene expression probe sets corresponding to 865 genes (Ingenuity Knowledge Base) were associated with the high responder analysis with the expression profile in the high responders differing from the rest (data not shown). Network analysis of the human interactome associated with these genes indicated that glucocorticoid, estrogen and insulin receptor signaling, and protein ubiquitination pathways were represented as top canonical pathways (*P*<0.001).

### Genetic markers associated with height change in girls with TS

Girls with TS had a median basal growth rate of 4 (*Q*1, 2; *Q*3, 6) cm/year, and then grew a median of 7.2 (*Q*1, 6.1; *Q*3, 9.1) cm over the first year. Two polymorphisms within two genes were found to be significantly associated with this growth response, assessed as a continuous trait (Table 2). These included the signaling molecule *KRAS* (MAPK pathway) and the pituitary transcription factor *LHX4*. As seen for GHD, the difference in growth between different alleles or genotypes was >1 cm over the first year.

*LHX4*, identified in the continuous trait analysis, was also found in the categorical analysis. In contrast to GHD, a further eight genes were added (Table 3): *IGFBP3* and *SOS1* (MAPK signaling), both found in GHD; *PIK3R3*, *PTPN1* and *PPP1CB* (all modulators of signaling); *CDK4* (a cell cycle regulator); *TGFB1* (a growth factor); and *MYOD1* (a muscle transcription factor). Importantly, the r-hGH doses between the high, intermediate and low responders did not differ (Table 4). Similar to GHD, the first PC based on the Tag SNPs separated those children from Asia from all other children, with the exception of one child (Fig. 1C). However, there was complete overlap in growth response between the groups (Fig. 1D).

A total of 1003 gene expression probe sets corresponding to 673 genes (Ingenuity Knowledge Base) were associated with first-year growth in the expression profiles for the low responder analysis (Fig. 2B): a distinct pattern of gene expression at baseline in the low responders compared with the other profiles was identified. A total of 700 gene expression probe sets corresponding to 506 genes (Ingenuity Knowledge Base) were associated with the high responder analysis with the expression profile in the high responders differing from the rest (data not shown). In contrast to GHD, no growth factor-related canonical pathways were represented by these genes.

### Integration of genetic and gene expression data

**Figure 3:**
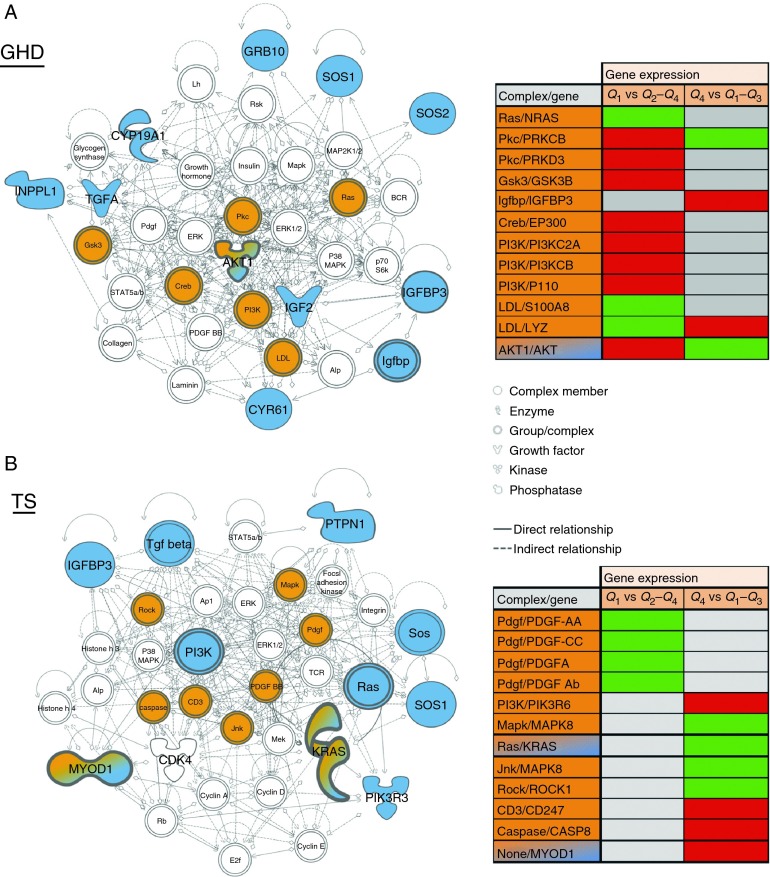
Biologic networks inferred from genes associated with growth response in children with (A) GH deficiency (GHD) and (B) Turner syndrome (TS). Blue shading indicates genes found to be associated with growth response in this study; orange shading indicates genes within the network associated with differences in baseline expression; orange/blue shading represents putative eQTLs, where there is both a genetic association and a change in gene expression; white shading represents genes in the inferred network, which have not been directly associated with growth response or gene expression difference. The tables show gene expression differences when comparing low growth response (quartile, *Q*1 vs *Q*2–*Q*4) and high growth response (quartile, *Q*4 vs *Q*1–*Q*3); green cells of the table represent downregulated gene expression; red cells of the table represent upregulated gene expression and gray cells of the table represent no change in gene expression.

To integrate the gene expression data with the genetic analysis, inferred networks were generated from the genetic data using the IPA functional association algorithm. In children with GHD, this procedure generated a network from all the associated genes (*AKT1*, *CYP19A1*, *CYR61*, *GRB10*, *IGF2*, *IGFBP3*, *INPPL1*, *SOS1*, *SOS2* and *TGFα* with the exception of *TP53*; Fig. 3A). In children with TS, an inferred network was generated from all the associated genes (*CDK4*, *IGFBP3*, *KRAS*, *MYOD1*, *PIK3R3*, *PTPN1* and *SOS1* with the exception of *LHX4* and *PPP1CB*; Fig. 3B). Gene expression data associated with first-year growth were mapped onto the inferred networks (Fig. 3A and B). One putative eQTL (a gene with both a genetic association and a change in expression) was identified in GHD, *AKT1*, and two putative eQTLs were identified in *TS*, *KRAS* and *MYOD1*. Other inferred network genes were also associated with changes in gene expression in either or both low and high quartiles of first-year growth (Fig. 3A and B), thus implying functional changes correlated with the genetic associations.

### *In silico* prediction of functional consequences of SNPs

Using the ENCODE database, in which transcription factor binding has been assessed in multiple cell lines by ChIP-seq, the SNPs in *IGFBP3*, *GRB10*, *CYP19A1* and *LHX4* fall within, or close to, transcription binding sites (Table 5).

## Discussion

GH is widely used to treat a range of growth disorders. Children who are sensitive to r-hGH in the first year of treatment and grow well are more likely to continue to gain height in the long-term (20, 21, 22). Identification of those who will be either sensitive or, more importantly, insensitive to r-hGH has important implications for counseling and clinical management. Current models to predict growth response to r-hGH over the first years of treatment have been based primarily on baseline auxologic characteristics and r-hGH dose, the latter being the only predictor that the treating physician can modulate (20, 21, 22); some models also include baseline IGF1 and IGFBP3 levels (both being GH-dependent biomarkers) and short-term change in bone markers (23, 24). In GHD, models based on auxology can predict up to 65% of the variability in the first year, and with the addition of biochemical markers, this is increased to 85%. In non-GH-deficient conditions, such models predict no more than 40–52% of the variability in first-year response; these predictions often have low accuracy (22). The PREDICT study is the first long-term study to evaluate the extent to which a range of genetic markers are associated with growth response.

For the DNA studies, a candidate gene approach was adopted, picking genes that affect the growth process not only directly but also indirectly by affecting metabolic pathways. Two very different growth disorders were examined, namely GHD, in which the cause was undefined, and TS, in which there was absence or structural abnormality of one X chromosome. The majority of the SNPs associated with first-year response to r-hGH differed between these conditions, implying that the genetic influences on the action of exogenous GH are not the same in the two conditions. In addition, this difference may relate to a ‘replacement’ GH dose in GHD vs a ‘pharmacologic’ dose in TS. However, SNPs in two genes, *IGFBP3* and *SOS1*, were common to both conditions, and these genes may have an impact on r-hGH sensitivity independent of the etiology of the growth disorder. Importantly, the differences in growth associated with the SNPs shown in Table 2 are of sufficient magnitude to be clinically important, accounting for ∼20% of the first-year response in this study.

Short stature associated with GHD without a defined etiology covers a broad spectrum, ranging from those with severe GHD, low IGF1 levels and very poor growth performance through to those with a mild impairment of GH secretion and low–normal IGF1, who in the majority of cases re-test as GH sufficient in late adolescence. This is the range of children who are treated as GHD, and if pharmacogenomics is going to aid the management of such children, then the genes associated with growth response to r-hGH must be significant across this broad diagnostic range. The PREDICT GHD cohort reflects this range of deficiency within the GH–IGF axis; children with severe GHD are represented, but also children with a modest impairment of GH secretion with normal IGF1 and IGFBP3 serum levels. It is also important to control for other confounding factors including age, r-hGH dose, pubertal progress, compliance and ethnic background. Therefore, we stratified the patients by age for the categorical analysis, using three age groups, each associated with quartiles of response. This identified some additional genes that were associated with growth response, more so in TS than GHD (Table 3). In the first month of this study, the dose of r-hGH to be given to children with GHD and TS was specified; thereafter, the clinicians determined the dose most appropriate for their patient. It was reassuring to find that the dose of r-hGH in both conditions was the same across the quartiles of growth response (Table 4), indicating that the dose was unlikely to be a major confounding factor. All were prepubertal at baseline, with approximately one-third entering the first stages of puberty by the end of the first year. This may have impacted modestly on growth rate in girls but not in boys. Compliance, as assessed by recall of injections given over the last month, was high in both conditions (mean ∼90%). It is very difficult to know the reliability of these data, and it is likely that this is a significant overestimation of compliance. It is well recognized that SNP frequencies vary between ethnicities, and this proved to be the case in this study with children from countries in South Asia separating very clearly from children from all other countries in the study when applying PCA to Tag SNPs (Fig. 1A and C). Nevertheless, this did not influence the magnitude of response to r-hGH between these two groups (Fig. 1B and D).

We also used a whole genome approach by analyzing gene expression profiles at baseline, using whole blood mRNA; the use of a PBMC model for variation of gene expression in response to r-hGH has previously been validated (11, 12). Gene expression signatures associated with both low and high growth responses in GHD and TS have been defined. In this study, genes related to growth factor action, signal transduction and cell cycle regulation were identified, emphasizing that many cellular processes affect response to r-hGH. In order to examine the potential functionality of the SNPs, we have looked at their proximity to transcription factor binding sites (Table 5). One of the *IGFBP3* SNPs (rs10255707) is located within an early growth response 1 (EGR1) binding site. EGR1 is a zinc-finger, nuclear protein that functions as a regulator of transcription, with studies suggesting that it is a cancer suppressor gene (gene ID: http://www.ncbi.nlm.nih.gov/gene/1958).

The principal carrier protein for IGF1, IGFBP3, whose expression is GH-dependent, was identified in both conditions (Tables 2 and 3B). IGF1 SNPs were not identified. This implies that IGFBP3 could have a greater overall impact on the variability of growth responses to r-hGH than IGF1. At the cellular level, IGFBP3 has both IGF1-dependent and direct, IGF1-independent effects on cell growth regulation (25). For the rs3110697 *IGFBP3* SNP, which is within 200 bp of STAT3 and EGR1 binding sites on the *IGFBP3* gene (Table 5), carriage of the G allele in GHD was associated with a high growth response (Table 2), but in girls with TS, carriage of the GG genotype was associated with a low growth response (Table 3B). These genotypes have been shown to associate with different serum levels of IGFBP3 in an adult multi-ethnic cohort (26); lowest levels were reported in those carrying the AA genotype, and 17% higher levels were found in those with a GG genotype. Thus, in GHD, a low growth response would associate with a relatively low IGFBP3 serum level, while in TS, a higher growth response would associate with low IGFBP3 levels. These apparently conflicting data are, however, supported by other clinical data; in a study assessing parameters that determine growth response on r-hGH treatment in GHD, IGFBP3 SDS was shown to have a positive relationship with change in height SDS (27). In a pharmacogenomic study examining the impact of an IGFBP3 SNP that also affects serum IGFBP3 in GHD, genotypes associated with higher IGFBP3 levels were associated with greater growth responses (28). In contrast, IGFBP3 has been identified as a negative factor in prediction models for response to r-hGH in children who are small for gestational age (23). In addition, in an *ex vivo* fibroblast model of growth factor action, TS cells produced more IGFBP3 than control cells in the basal state, but generated less IGFBP3 in response to IGF1 stimulation, implying that higher IGFBP3 levels in the media around these cells were inhibiting IGF1 action (29). Therefore, the influence of IGFBP3 appears to be disease dependent, and this is reflected in the divergent growth responses associated with the same IGFBP3 SNP in GHD and TS. These differing associations may be due to the different r-hGH doses received by patients with GHD and TS (larger in TS), as well as differences in growth plate resistance to GH and/or IGF1.

In patients with GHD, six SNPs in *GRB10* were associated with growth response (Tables 2 and 3A). GRB10 interacts with insulin and IGF1 receptors; its overexpression inhibits tyrosine kinase receptors leading to growth suppression (gene ID: http://www.ncbi.nlm.nih.gov/gene/2887). Two of the SNPs are within 200 bp of transcription factor binding sites and would be predicted to have an effect on transcriptional regulation (Table 5). The SNP in the *CYP19A1* gene is located within a Fos–Jun site and has been shown to impact transcriptional activity – the C allele had 60% higher promoter activity than the A allele (30). Growth rate in GHD was lower in TT homozygotes, implying that lower aromatase activity would associate with poorer growth responses. This observation suggests that even before puberty, low levels of estrogen may contribute to growth response to r-hGH.

In girls with TS, the transcription factor LHX4, which when mutated is associated with multiple pituitary hormone deficiencies, was associated with growth response. Girls with TS have normal endogenous GH secretory capacity. However, this SNP is located within 200 bp of a CTCF-binding site; CTCF is another zinc-finger protein known to regulate transcription (Table 5). This association may suggest a role for regulation of pituitary hormones in first-year response to r-hGH treatment in TS. In addition to *IGFBP3*, SNPs identified in both conditions included genes within the MAPK pathway – *SOS1* and *SOS2* in GHD, and *SOS1* and *KRAS* in TS. *SOS1* and *KRAS*, but not *SOS2*, have been implicated in human rasopathies, including mutations in *SOS1* and *KRAS* in Noonan syndrome and *KRAS* mutations in cardiofaciocutaneous syndrome (31). This indicates that the MAPK pathway is a key regulator of r-hGH responsiveness.

Using a network approach to analysis, we have shown that genes within networks associated with growth response are differentially expressed between high and low responders to r-hGH in both TS and GHD (Fig. 3). Importantly, the latter included three genes containing SNPs associated with growth response (one in GHD, two in TS), demonstrating that these SNPs are associated with a change in expression in that gene.

This study has identified potential genetic markers and expression profiles for growth response to r-hGH in patients with GHD or TS, and has broadened considerably the spectrum of genes associated with GH action. These findings must be validated in independent cohorts, including the full range of growth disorders treated with r-hGH. These results indicate that pharmacogenomics could have a role to play in a personalized strategy for managing r-hGH treatment in children.

## Supplementary data

This is linked to the online version of the paper at http://dx.doi.org/10.1530/EJE-13-0069.

Supplementary Table

## Figures and Tables

**Table 1:** Patient demographic data at baseline. Data are number (%) or median (*Q*1 and *Q*3). Minimum and maximum values are shown in brackets.

**Per-protocol population at year 1**	**GHD** (*n*=115)	**Min, max values**	**TS** (*n*=67)	**Min, max values**
Male (%)	69 (60)^a^		0 (0)	
Female (%)	46 (40)^a^		67 (100)^a^	
Age (years)	9.8 (6.8, 11.3)	2, 15	9.1 (6.3, 11.8)	3, 16
Mid-parental height SDS	−0.8 (−1.6, −0.1)	−4, +2	−0.1 (−0.9, 0.6)	−4, +2
Height SDS	−2.1 (−2.7, −1.7)	−6.5, −0.3	−2.4 (−3.1, −1.5)	−5.4, −0.2
BMI SDS	−0.3 (−1.0, +0.5)	−3, +10.3	+0.6 (−0.3, +1.6)	−2.2, +4.8
GH peak response (μg/l)	4.1 (2.6, 5.6)	0, 9	NA	
IGF1 SDS	−1.8 (−2.8, −0.9)	−7.7, +1.1	−1.2 (−2.1, −0.5)	−4.5, +1.7
IGFBP3 SDS	−0.2 (−1.1, +0.5)	−5.3, +2.2	+0.3 (−0.2, +0.7)	−2.3, +2.1

GHD, GH deficiency; IGFBP3, insulin-like growth factor binding protein 3; NA, not applicable; *Q*, quartile; TS, Turner syndrome.

a All were Tanner stage 1 at baseline. Thirty-seven children with GHD and 12 girls with TS entered puberty over the first year. DNA samples for genotyping were available for 110 patients with GHD and 60 girls with TS.

**Table 2:** Single nucleotide polymorphisms (SNPs) in genes associated with first-year growth response in (A) GHD and (B) TS. Mean height change for genotypes is shown. More than one SNP was associated with growth for *IGFBP3, GRB10, TGFα* and *INPPL1*.

**Gene**	**Function**	**SNP ID**	**Model**	**Genotype** (*n*)	**Mean height change**, cm (s.d.)	**Nonadjusted *P* value**	**Adjusted *P* value**
GHD							
*GRB10*	Insulin and IGF1 signaling	rs1024531	Genotype	GG (5)	6.2 (1.4)	0.0005	0.01
				GA (45)	8.0 (2.0)		
				AA (60)	9.4 (1.9)		
		rs1024531	Allele^a^	AA (60)	9.4 (1.9)	0.0006	0.0121
				GG and GA (50)	7.9 (2.0)		
		rs12536500	Allele^a^	CC (70)	9.2 (2.0)	0.001	0.0253
				TT and TC (40)	7.9 (2.0)		
		rs933360	Allele^a^	TT (71)	9.2 (2.2)	0.002	0.0448
		TC and CC (39)	8.0 (1.6)		
*IGFBP3*	Binding protein for IGFs	rs3110697	Allele^b^	AA (18)	7.5 (2.0)	0.002	0.0117
		GG and GA (91)	9.0 (2.1)		
		rs10255707	Allele^b^	CC and CT (92)	9.0 (2.1)	0.006	0.0278
				TT (13)	7.3 (2.0)		
		rs3110697	Genotype	AA (18)	7.4 (2.0)	0.009	0.0442
				AG (42)	9.2 (2.1)		
		GG (49)	8.9 (2.0)		
*TGFα*	Growth factor	rs958686	Genotype	GG (14)	7.4 (1.0)	0.002	0.0351
				GC (60)	8.6 (2.1)		
		CC (36)	9.6 (2.3)		
		rs958686	Allele^a^	CC (36)	9.6 (2.3)	0.002	0.046
		GG and GC (74)	8.0 (2.0)		
*CYP19A1*	Aromatase enzyme for estrogen synthesis	rs10459592	Allele^a^	GG (29)	10 (2.2)	0.003	0.043
					
		TT and TG (81)	8 (1.9)		
*SOS1*	MAPK signaling pathway	rs2888586	Allele^a^	CC (33)	7.8 (1.8)	0.0095	0.0476
		TT and CC (77)	9.0 (2.0)		
*TP53*	Cell cycle regulation	rs2909430	Allele^a^	TT (77)	9.1 (2.1)	0.014	0.0414
		TC and CC (33)	8.0 (1.8)		
*INPPL1*	Regulation of insulin and growth factor receptor signaling	rs2276048	Allele^a^	AA (72)	8.4 (1.9)	0.0254	0.0254
					
				AG and GG (38)	9.5 (2.3)		
		rs2276048	Genotype	AA (72)	8.4 (1.9)	0.0497	0.0497
				AG (34)	9.4 (2.4)		
			GG (4)	10.1 (1.3)		
TS						
*LHX4*	Pituitary transcription factor	rs3845395	Allele^a^	GG (31)	6.9 (1.4)	0.002	0.0485
		GC and CC (29)	8.3 (1.8)		
*KRAS*	MAPK signaling pathway	rs12579073	Genotype	AA (22)	8.0 (1.7)	0.008	0.0461
				AC (24)	6.8 (1.4)		
		CC (14)	8.4 (1.9)		

a Allele, major allele recessive.

b Allele, major allele dominant.

**Table 3:** Genes identified using the categorical model, based on quartiles (*Q*) of growth response and age bands of (A) patients with GHD and (B) girls with TS.

**Gene**	**Function**	**Marker**	**Categorical model**	**Categorical nonadjusted** *P* value	**Categorical adjusted** *P* value	**Relative risk**	**95% CI relative risk**	**Category 1**	**Category 2**	**Genotype marker for category 1**	**Genotype marker for category 2**
(A) GHD											
*GRB10*	Insulin and IGF1 signaling	rs1014384	Dominant	0.0012	0.0245	3.6	2.15, 5.94	L	I + H	GG	AA and AG
*GRB10*^a^	Insulin and IGF1 signaling	rs933360	Recessive	0.0012	0.0259	4.8	1.54, 14.73	H	I + L	TT	CC and TC
*GRB10*	Insulin and IGF1 signaling	rs4521715	Recessive	0.0013	0.0266	4.6	1.48, 14.14	H	I + L	AA	GG and AG
*CYP19A1*^a^	Aromatase enzyme	rs10459592	Recessive	0.0030	0.0455	2.6	1.44, 4.71	H	I + L	GG	TT and TG
*SOS2*	MAPK signaling	rs13379306	Recessive	0.0060	0.0482	2.4	1.31, 4.49	L	I + H	AA and AC	CC
*CYR61*	Secreted protein, IGFBP10	rs9658584	Recessive	0.0083	0.0167	2.7	1.26, 5.80	H	I + L	GG	CC and CG
*SOS1*^a^	MAPK signaling	rs2888586	Recessive	0.0086	0.0429	3.7	1.21, 11.42	H	I + L	TT and TC	CC
*IGF2*	Growth factor	rs3213221	Dominant	0.0138	0.0414	2.5	1.36, 4.45	H	I + L	CC	GG and CG
*AKT1*	Activated by PI3K	rs1130214	Recessive	0.0290	0.0290	2.3	1.10, 4.68	L	I + H	AA and AC	CC
*INPPL1*^a^	Regulation of insulin and growth factor signaling	rs2276048	Recessive	0.0392	0.0392	2.0	1.10, 3.75	H	I + L	GG and AG	AA
(B) TS											
*LHX4*^a^	Transcription factor	rs3845395	Recessive	0.0003	0.0067	7.5	1.86, 30.11	H	I + L	CC and GC	GG
*LHX4*	Transcription factor	rs4652492	Recessive	0.0013	0.0275	NA	NA	H	I + L	AA and AG	GG
*PTPN1*	Protein tyrosine phosphatase (in insulin and JAK2 signaling)	rs13041704	Dominant	0.0037	0.0261	4.7	2.83, 7.71	L	I + H	CC	AA and AC
*PIK3R3*	Regulatory subunit of PI3K	rs809775	Recessive	0.006	0.0181	3.3	1.51, 7.14	L	I + H	TT	AA and AT
*PPP1CB*	Catalytic subunit of protein phosphatase 1	rs6725177	Recessive	0.006	0.0299	3.3	1.41, 7.86	L	I + H	CC	GG and GC
*CDK4*	Cell cycle regulator	rs2069502	Recessive	0.0073	0.0146	5.0	1.25, 20.07	H	I + L	TT and TC	CC
*SOS1*	MAPK signaling	rs2168043	Recessive	0.0079	0.0394	3.3	1.44, 7.33	H	I + L	AA and AC	CC
*IGFBP3*	IGF binding protein	rs3110697	Recessive	0.0084	0.0421	3.3	1.31, 8.30	L	I + H	GG	AA and AG
*TGFB1*	Growth factor	rs4803455	Recessive	0.0126	0.0379	NA	NA	H	I + L	AA and AC	CC
*MYOD1*	Transcription factor (in muscle)	rs3911833	Recessive	0.0476	0.0476	5.2	0.74, 36.47	L	I + H	CC	TT and TC

GHD, GH deficiency; H, high responder (≥*Q*3); I, intermediate responder (>*Q*1, <*Q*3); L, low responder (≤*Q*1); NA, not applicable; *Q*, quartile; TS, Turner syndrome.

a Genes also identified in the continuous analysis.

**Table 4:** Mean r-hGH doses in GHD and TS in those with high (≥*Q*3), intermediate (>*Q*1, <*Q*3) or low responses (≤*Q*1).

	**High**	**Intermediate**	**Low**
GHD			
Mean r-hGH dose (μg/kg per day)	0.0341	0.0350	0.0335
95% CI	0.0317–0.0365	0.0330–0.0371	0.0307–0.0364
Minimum, maximum values	0.0243, 0.0524	0.0164, 0.0563	0.0218, 0.0621
TS			
Mean r-hGH dose (μg/kg per day)	0.0469	0.0482	0.0490
95% CI	0.0396–0.0541	0.0431–0.0533	0.0440–0.0517
Minimum, maximum values	0.0416, 0.0563	0.0375, 0.0607	0.0347, 0.0581

GHD, GH deficiency; r-hGH, recombinant human GH; *Q*, quartile; TS, Turner syndrome.

**Table 5:** Physical association of significant SNPs in continuous and categorical analyses in (A) GHD and (B) TS to known transcription factor binding sites.

**Gene**	**SNP**	**Transcription factor binding sites**^a^
(A) GHD		
*GRB10*	rs1024531	Within 200 bp of ‘Neurone-restrictive Silencer factor’ (NRSF) site
rs12536500	Within 200 bp of ‘Upstream Regulatory factor’ (USF) half site
*CYP19A1*	rs10459592	Within Fos/Jun site
*IGFBP3*	rs3110697^b^	Within 200 bp of STAT3 and EGR1 sites
rs10255707	Within EGR1 site
*CYR61*	rs9658584	Within P300/JUN/KAP1 sites
*AKT1*	rs1130214	Within CTCF/Rad21 sites
(B) TS		
*LHX4*	rs3845395	Within 200 bp of CTCF site
rs4652492	Within 200 bp of CTCF/Rad21 sites
*PIK3R3*	rs809775	Within 200 bp of STAT3 site
*CDK4*	rs2069502	Within 200 bp of CTCF site
*SOS1*	rs2168043	Within CEBPB site
*IGFBP3*	rs3110697^b^	Within 200 bp of STAT3 and EGR1 sites

Bp, base pairs; GHD, GH deficiency; SNP, single nucleotide polymorphism; TS, Turner syndrome.

a These data have been derived from many different cell lines of which transcription factors and their binding sites responsible for modulating gene transcription, as identified by ChIP-seq, are listed in the ENCODE database (http://genome.ucsc.edu/ENCODE/). The SNPs identified in this study lie in or near these binding sites.

b Same SNP found in both GHD and TS.
