# Supplementary material for: A pharmacogenomic approach to the treatment of children with GH deficiency or Turner syndrome
Source: Eur J Endocrinol. 2013 May 24;169(3):277–89. doi: 10.1530/EJE-13-0069 (PMC3731924; doi:10.1530/EJE-13-0069)
Supplement: Supplementary Table [file supp_169_3_277__index.html]

Supplementary Table 

# A pharmacogenomic approach to the treatment of children with GH deficiency or Turner syndrome

## Supplementary Table

**Files in this Data Supplement:**

- Supplementary Appendix - List of candidate genes used in this analysis. (PDF 50 KB)
