# Supplementary material for: A pharmacogenomic approach to the treatment of children with GH deficiency or Turner syndrome
Source: Eur J Endocrinol. 2013 May 24;169(3):277–89. doi: 10.1530/EJE-13-0069 (PMC3731924; doi:10.1530/EJE-13-0069)
Supplement: Supplementary Table [file supp_EJE-13-0069_Supplementary_table_1.pdf]

SUPPLEMENTARY APPENDIX

Online Supplementary Table 1. List of candidate genes used in this analysis

GH and IGF-1-related genes	IGF-1/IGF-1R signalling	Insulin-related	Bone metabolism	Oncogenes	Inflammatory genes	Pituitary transcription factors	Cell growth
GHR	*IGF2*	*INS (*insulin*)*§	*ESR1*	*FOS*	*IL-4*	*LHX4*	*FGF3*
GHRHR	*IGF2R*	*INSR*	*AR*	*TGFB1*	*IL-6*	*PROP1*	*SOS2*‡
STAT cluster	*IGFBP2*	*IRS1*	*RARα*	*TGFB2*	*GATA1*	*POU1F1*	*GRB2*
SOCS2	*IGFBP3*	*IRS2*	*RARβ*	*TP53*	*SH2B2*		
JAK2	*ID1*	*IRS4*	*PRKCA*	*MYC*			
IGF-1	*GAB1*	*SHC1*	*MYOD1*	*RB1*			
IGF1R	*PI3* kinase genes*‡	*SOS1*	*CDKN1A (*p21*)*	*WT1*			

IGFALS[1]	*SHC1*	*PPP1CA*[1]	*CYP19A1*	*BCL2*			
	IGFBP1	*PPP1CB*	*SHOX*	*CDK2*			
	PDGFRB	*PPP1CC*	*SHOX2*[§]	*CDK4*			
	ARRB1	*PTPN1*		*CDK6*			
	GRB10	*PDK1*		*EGFR*			
	PTPNS1 (*SHPS1/SIRPA*)	*AKT1*		*TGFA*			
	CYR61 (*IGFBP10*)	*AKT2*		*CCND1*			
		PPARG		*CCND2*			
		PPARGC1A		*CCND3*			
		GLUT1 (*SLC2A1*)		*KRAS*			

		GLUT4 (*SLC2A4)*					
		LEP					
		LEPR					
		HRAS[†]					
		NRAS[†]					
		INPPL1 (*SHIP2)*					
		SREBF1					
		ACDC (*ADIPOQ)*					

*Includes the following genes: *PIK3CA*, *PIK3CB*, *PIK3CD, PIK3CG*, *PIK3R1*, *PIK3R2*, *PIK3R3*.

[†]Gene also involved in bone metabolism.

[‡]Gene also involved in insulin-related pathways.

[§]Only analyzed in GHD samples.

[l]Only analyzed in TS samples.

GH, growth hormone; GHD, growth hormone deficiency; IGF, insulin-like growth factor; IGF-1R, insulin-like growth factor 1 receptor; TS, Turner syndrome.
